# On Certain Abuses of Caustics

**Published:** 1864-08

**Authors:** James Morton

**Affiliations:** Lecturer on Materia Medica, Anderson’s University, and Surgeon to the Glasgow Royal Infirmary


### MISCELLANEOUS.

### ON CERTAIN ABUSES OF CAUSTICS.

By Dr. James Morton, Lecturer on Materia Medica, Anderson's University, and Surgeon to the Glasgow Royal Infirmary.

[Dr. Morton considers that the use of caustics is often abused, and he thinks such errors may frequently be traced to directions contained in the works of some of the leading writers of the day. We find them frequently employed in some diseases affecting the whole system, over which caustics, as such, can have no influence whatever.]

In common inflammation of the fauces, popularly known as sore-throat, and usually ascribed to cold as a cause, and whose symptoms I do not take time to enumerate, presuming that no one can mistake it, surely the employment of caustics cannot be said to be requisite. But besides being unnecessary, there is the additional objection that it is the means of inflicting a very considerable amount of pain. The disease will speedily disappear, if left to itself, or treated by soothing agents. Should suppuration take place, the possibility of the caustic application producing an earlier evacuation of the pus must be admitted. This, however, must be regarded as an accidental circumstance, as the caustic is not employed with this intention.

Very much the same may be said in respect to ulcerated sore-throat, except that in addition some mild alterative may be required, and a caustic application can only rarely, if ever, be necessary to repress prurient granulations, as in other parts of the body.

In regard to the three eruptive diseases, measles, scarlet fever, and small pox, in all of which the throat is so apt to become affected, it is difficult to speak so as to avoid misconstruction. When a slough forms upon the fauces, the part is often diligently assailed by the over-zealous practitioner, in the hope of thereby arresting the sloughing process; and when it ceases to extend, he is thought to have succeeded, and he plumes himself and is praised by others accordingly. I have no hesitation in affirming that this gratulation is often misplaced, and that the slough would have been smaller, and would more speedily have disappeared, had no caustic application been resorted to. This is not unfrequently exemplified in the treatment of scarlet fever, in which the mucus surface of the throat often suffers severely. Almost every one who has seen much practice must have witnessed cases of this severe throat complication occurring in children, not infants, who have obstinately and perseveringly, and with success, resisted all attempts to cauterise their throats, or even to touch them. In very many instances these do as well as, or better than those who may have been subjected to the ordeal; and the former are often as severe in the character of the attack as the latter. The more rarely, therefore, that we employ caustics in such cases, the better for our patients, and the more pleasant will be our treatment. All the local applications may be of a soothing nature, and it is not our present duty to discuss the constitutional treatment. (It may here be proper to remark also, that the erosive treatment of small-pox by nitrate of silver is a topic foreign to the object of this paper.) Some have an idea that active treatment of the local complication has a powerful influence over the constitutional affection; or, in other words, that the speedy removal by caustic applications of the morbid exudations from the situations upon which the disease seems to fasten in its greatest intensity, is of the very greatest effect in counteracting the deleterious action of the morbid poison upon the general system. This notion (it is scarcely entitled to be called an opinion) will not be maintained by many, and is such an untenable position that it does not seem to be necessary to attack it. An opposite mode of reasoning is taken by the majority, viz: that the severity of the systemic poisoning has much to do with the intensity of the local manifestations of it.

After what has now been said with more especial reference to scarlatina, it is not requisite to say much respecting measles and small-pox. Affections of the throat are not so common in these two varieties of the exanthemata, and the objectioas already urged may apply to rubeolous and variolous cases in which the throat is found to suffer.

In the disease now styled diphtheria the use of caustics, as they are too often employed, is, in my opinion, productive of the most disastrous consequences. All the symptoms of this malady indicate the presence of a general toxic agent, probably an epidemic poison, and the prominent symptoms are those of debility, the throat affection coming on insidiously, often unperceived for a time, often with little or no pain, and a slight degree of swelling, though in some cases the tumefaction in and around the throat is very considerable. The chief indication is to support the strength; another important though subsidiary one ought to be, to avoid measures calculated to add to the existing local complications. It is a custom with some, I hope not with many, to divest the fauces of the whitish leathery covering which forms upon them, literally to dissect it off, and then to apply the solid nitrate of silver or some liquid caustic freely; and not only so, but to repeat this process daily, or as often as the adventitious membrane reforms, and in the belief that they are only doing what is absolutely necessary towards giving their patient the best chance of recovery. This line of practice I regard as a woeful mistake. It seems to me that, by so acting, the surgeon is diligently endeavoring to undo all that nature is attempting to effect towards a spontaneous cure of the malady. Surely no one imagines, that by tearing the exudation off the fauces, he will prevent its extension into the larynx and trachea. Such a procedure seems to me more likely to promote the dangerous progress of the false membrane, to use a phraseology now somewhat antiquated. To prevent misconstruction, let me add that no one can object to the removal of sloughy matters flapping loosely in the pharynx.

A recent writer has said that the more copiously lymph exudes upon the fauces and larynx, the less likely is it to be deposited along the interior of the bronchial tubes in croup and diphtheria; and the inference from that is that in such instances tracheotomy is more likely to succeed. This assertion we may not all admit, and, at all events, it has not been established as a fact; but though it were, caustics are not used for the purpose of promoting the deposition of lymph, neither will their free application render the success of tracheotomy more probable.

In the belief, then, that caustics in all forms are injurious in diphtheria, I would venture to recommend their complete abandonment in the treatment of this peculiar but perilous disease.

However improbable it may be that I shall be met with the objection that my remarks are directed against a practice which does not now obtain, it may be right to state that any one who glances at the weekly and monthly medical journals of the day, will at once be satisfied that such is not the case. In the Edinburgh Medical Journal for October last, there is a lengthy article upon diphtheria, the writer of which advocates the vigorous use of caustics, even to the dropping of it into the larynx by a tube, and congratulates himself on thereby obtaining complete command of the symptoms. It is also worthy of remark that this practitioner uses iodide of potassium in ordinary doses, a mode of treatment previously proposed and employed by Mr. Wade, of Birmingham. The cases thus treated did not speedily arrive at convalescence—not so speedily as most recoverable cases of diphtheria usually do—and it may possibly yet occur to the writer that he may accelerate his cures by confining his medication to the latter remedy, and entirely excluding the caustics. The same writer mentions a family in which he was attending one diphtheritic patient, where there were other five inmates similarly affected in some degree; and when describing their symptoms and treatment, he says: "I had cauterised and sponged with the caustic solution their throats daily, and I had ordered to be taken, for three or four days, ten drops of the tinc. mur. ferri twice daily. Nevertheless, the inflammation of the throat and the effusion of lymph kept slowly but steadily advancing." In a subsequent page, when speaking generally of treatment, he remarks: "If the patient, when I was called, was free from fever, but had the throat affected, I contented myself with touching the lymph with the caustic, and sponging the throat, fauces, and glottis with the same solution, and gave the sol. iod. pot. from three times daily to every two hours, according to the urgency of the case." And again, "Where there was the slightest hoarseness, I never failed also at once to drop the caustic solution into the windpipe." Surely a striking example of the *nimia diligentia medicinæ*, and the steady advance of the inflammation and effusion would surprise no one but the writer.

A careful attention to the current medical literature of the day, will convince almost any one that in some cases recovery is not to be ascribed to the local means employed, but rather that it takes place in favorable cases in spite of these means, which can only have the effect of protracting the period of disease, or delaying convalescence. It is perfectly well known to me, that in holding such opinions I do not stand alone, though I fear they are held and acted upon by but a few, not by a minority.

While writing this paper my attention was directed, as previously noted, to a pamphlet entitled "Notes on Clinical Medicine," by Dr. W. F. Wade, of Birmingham, the first part of which relates to diphtheria; and after stating that "local treatment exerts no known influence upon the general course of specific fevers," he continues in a succeeding page as follows:

"It is contrary to the ordinary rules of our art to interfere with the local development of blood poisons, except for special reasons."

"The faucial exudation of diphtheria is to be considered as the local manifestation of a general disease."

"Interference with it will not prevent its reproduction, nor will it prevent laryngeal complication, nor will it prevent the supervention of grave constitutional disorder. It is, besides, exceedingly irksome to young patients."

"We are justified in interfering with the throat exudation when there is excessive fœtor, or when it is so copious as to interfere with respiration or deglutition—not otherwise."

These opinions coincide so exactly with my own in respect to local management, that I have taken the liberty of quoting them; and it may be added by the way that Dr. Wade recommends, for constitutional treatment, iodide of potassium, iodide of iron, and bichloride of mercury, with bark, as eliminants of the blood-poison.

Lastly, in reference to syphilis, less requires to be said; for, while it can not be doubted that caustics are still too frequently employed, and productive of considerable mischief, yet it must also be remembered that their frequent and indiscriminate employment is not sanctioned by the best authorities on the treatment of this disease.—*Glasgow Medical Journal, Jan.* 1864, *p.* 409. *Braithwaite's Retrospect.*

